# Species distribution and introgressive hybridization of two *Avicennia* species from the Western Hemisphere unveiled by phylogeographic patterns

**DOI:** 10.1186/s12862-015-0343-z

**Published:** 2015-04-10

**Authors:** Gustavo M Mori, Maria I Zucchi, Iracilda Sampaio, Anete P Souza

**Affiliations:** Centro de Biologia Molecular e Engenharia Genética, Universidade Estadual de Campinas, CEP 13083-875, CP 6010 Campinas, São Paulo Brazil; Pólo Centro Sul, Agência Paulista de Tecnologia dos Agronegócios. Piracicaba, CEP 13400-970, São Paulo, Brazil; Universidade Federal do Pará, Campus de Bragança, Instituto de Estudos Costeiros, CEP 68600-000, Bragança, Pará Brazil; Departamento de Biologia Vegetal, Instituto de Biologia, Universidade Estadual de Campinas, CEP 13083-862, Campinas, São Paulo Brazil

**Keywords:** Phylogeography, Introgression, cpDNA, nDNA, Transoceanic dispersal

## Abstract

**Background:**

Mangrove plants grow in the intertidal zone in tropical and subtropical regions worldwide. The global latitudinal distribution of the mangrove is mainly influenced by climatic and oceanographic features. Because of current climate changes, poleward range expansions have been reported for the major biogeographic regions of mangrove forests in the Western and Eastern Hemispheres. There is evidence that mangrove forests also responded similarly after the last glaciation by expanding their ranges. In this context, the use of genetic tools is an informative approach for understanding how historical processes and factors impact the distribution of mangrove species. We investigated the phylogeographic patterns of two *Avicennia* species, *A. germinans* and *A. schaueriana,* from the Western Hemisphere using nuclear and chloroplast DNA markers.

**Results:**

Our results indicate that, although *Avicennia bicolor, A. germinans* and *A. schaueriana* are independent lineages, hybridization between *A. schaueriana* and *A. germinans* is a relevant evolutionary process. Our findings also reinforce the role of long-distance dispersal in widespread mangrove species such as *A. germinans*, for which we observed signs of transatlantic dispersal, a process that has, most likely, contributed to the breadth of the distribution of *A. germinans*. However, along the southern coast of South America, *A. schaueriana* is the only representative of the genus. The distribution patterns of *A. germinans* and *A. schaueriana* are explained by their different responses to past climate changes and by the unequal historical effectiveness of relative gene flow by propagules and pollen.

**Conclusions:**

We observed that *A. bicolor, A. germinans* and *A. schaueriana* are three evolutionary lineages that present historical and ongoing hybridization on the American continent. We also inferred a new evidence of transatlantic dispersal for *A. germinans*, which may have contributed to its widespread distribution. Despite the generally wider distribution of *A. germinans,* only *A. schaueriana* is found in southern South America, which may be explained by the different demographic histories of these two species and the larger proportion of gene flow produced by propagules rather than pollen in *A. schaueriana*. These results highlight that these species responded in different ways to past events, indicating that such differences may also occur in the currently changing world.

**Electronic supplementary material:**

The online version of this article (doi:10.1186/s12862-015-0343-z) contains supplementary material, which is available to authorized users.

## Background

Mangrove forests are unique tree communities that occupy narrow elevation ranges within the intertidal zones of tropical and subtropical regions. Compared with tropical and subtropical terrestrial plant communities, the few species that occupy these forests are characterized by physiological and ecological traits that make them highly adapted to the coastal environment [1]. The latitudinal distribution of these organisms is mainly determined by both climatic and oceanographic features, including the occurrence of frosts, air and sea surface temperature, precipitation and a suitable intertidal habitat [2-6]. In the context of recent global climate change, there is evidence that these species are currently expanding their geographic distributions poleward within the two major mangrove biogeographic regions: the Atlantic Caribbean East-Pacific region (ACEP) [5,7-10] and the Indo West-Pacific region (IWP) [11-14]. As would be expected from this evidence of current expansion, palynological and stratigraphic data indicate that in the recent past (from the late Holocene and Pleistocene), climatic alterations influenced the worldwide distribution of mangroves [6,15,16]. The use of genetic data is an interesting approach to complement the palynologic and stratigraphic methods and to shed light on how the distribution of mangrove trees has changed over time and space.

**Figure 1:**
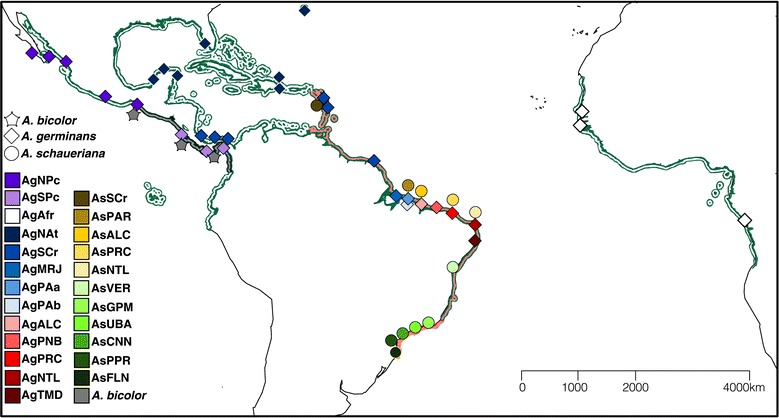
**Map showing sampling design.** Locations of the samples of *Avicennia bicolor, A. germinans* and *A. schaueriana* (represented by the shape of the polygons) across the Western Hemisphere. The color of each polygon refers to the geographic region where the sample was obtained according to Additional file 1. The current geographic distributions of *A. bicolor, A. germinans* and *A. schaueriana* are shown along the coastlines of the continents in gray, green and red, respectively; the zones of sympatry between the species are denoted by overlapping colors.

In the ACEP region, for example, *Rhizophora mangle* L. (Rhizophoraceae) has expanded its distribution southward along the Brazilian coast since the last glacial maximum (LGM) [17]. Furthermore, evidence shows that in the northern part of the ACEP biogeographic region, *Avicennia germinans* L. (Acanthaceae) populations have expanded their ranges northward since the LGM [18,19]. For both species, there is evidence of long-distance dispersal (LDD) [18,20], reinforcing the key role of dispersal as an important biogeographic mechanism in the process of population extinction and posterior recolonization [18]. To expand on these efforts and to better understand how mangrove forests have been changing in response to historical factors and processes, we studied the phylogeographic patterns of two *Avicennia* species of the Western Hemisphere: *Avicennia germinans* and *A. schaueriana* Moldenke. The former is a widespread species found throughout most of the ACEP region, whereas the latter is restricted to the Atlantic coast of South America and the southern Caribbean [1,21,22] (Figure 1).

The genetic structure of these species is influenced by intrinsic factors, such as mixed mating systems, biparental inbreeding, ongoing hybridization, and rate of gene flow through pollen and propagule dispersal [18,23-26], and by extrinsic factors, such as marine currents and tidal patterns [26]. The combination of this complex set of ecological features that shape the genetic diversity of *A. germinans* and *A. schaueriana* and historical processes, such as global climate changes after the LGM, may shed light on possible explanations for the current distribution of mangrove species in South America [1,21,22].

Our main objective was to evaluate the genetic variation in the *Avicennia* species extending across nearly the entire mangrove forest distribution in the Western Hemisphere and to unveil some evolutionary processes that could have shaped this genetic diversity. Through intensive and extensive sampling along the Brazilian coastline, coupled with samples from the Pacific coastline areas of Central America, the Caribbean and West Africa [18,23] and the sequencing of chloroplast and nuclear DNA (cpDNA and nDNA, respectively) markers, we provide a large-scale assessment of the genetic variation of *Avicennia* covering nearly the entire ACEP region. This strategy enabled us to gain a broad molecular perspective on the evolutionary history of the genus*,* including the three species found in this biogeographic region: *A. germinans, A. schaueriana* and *A. bicolor* Standl.; the distribution of *A. bicolor* is restricted to the Pacific coast of Central America (Figure 1). This distribution of species is particularly interesting because there is evidence to suggest that there has been ancient hybridization between *A. germinans* and *A. bicolor* within their zone of sympatry [23] and an ongoing and unidirectional introgression process between *A. germinans* and *A. schaueriana* on the northern coast of South America [23,26]. We also studied the geographic distribution of the genetic diversity across the ACEP region to better comprehend the previously described complex interplay between the intrinsic and extrinsic factors that are influencing the neutral genetic variation of the species [26]. We then evaluated the evidence for the historical demographic fluctuations of *A. germinans* and *A. schaueriana* and the historical ecological differences between them to explain the current pattern of species distribution along the South American continent.

## Methods

### Plant material

We sampled 138 *A. germinans* and 193 *A. schaueriana* individuals from 11 locations along the Brazilian coastline; the samples were georeferenced using a global positioning system (Garmin 76CSx, considering the WGS84 standard) (Figure 1 and Additional file 1). For simplicity, each sample is henceforth denoted as in Additional file 1, with Ag and As indicating *A. germinans* and *A. schaueriana*, respectively, followed by a three-letter abbreviation corresponding to the site where the individuals were obtained. These species were identified in the field based both on their floral structures and vegetative branches [1] to minimize the chances of misidentification. Voucher specimens from every site, except for Alcântara, Maranhão, were deposited in the EMBRAPA Amazônia Oriental (IAN) and University of Campinas (UEC) herbaria.

From each individual plant, we selected young and visually healthy leaves and maintained them in sealed bags containing silica gel; the samples were kept in the bags until being lyophilized and then stored at −20°C. The desiccated material was then ground into a fine powder using liquid nitrogen, and the resulting powder was used to isolate total DNA via a cetyltrimethylammonium bromide protocol.

### Genetic analyses

To evaluate the distribution of the genetic variation, we sequenced two intergenic spacers of the chloroplastidial genome and one region of the nuclear ribosomal internal transcribed spacer (ITS). The *trn*D-*trn*T and *trn*H-*trn*K spacers of the cpDNA were amplified using the previously described DT and HK primer sets [27], and the polymerase chain reaction (PCR) amplification of the ITS region was performed using the previously described LEU1 and ITS4 primers [28]. The sequencing reactions were performed using two primers specific to the *trn*D-*trn*T and ITS markers, and the *trn*H-*trn*K locus was partially sequenced with primer H, as performed by Nettel and colleagues [23]. The sequences were deposited in the DNA Data bank of Japan (Additional file 1). To augment the geographic distribution of our study and to include samples of *A. bicolor* and of two *Avicennia* species from the IWP biogeographic region, *A. alba* Blume and *A. marina* (Forssk.) Vierh., we also included previously analyzed sequences [18,23]. For population-level analyses, we only considered samples with eight or more individuals and used the chloroplast and nuclear markers that were available from each geographic region. Due to the differences in the publicly available sequences of previous studies [18,23], we considered different numbers of individuals for the cpDNA and nDNA markers (see Additional file 1).

We assembled and manually verified the chromatograms using CLC Genomics Workbench 4.9 software (CLC Bio). When we detected evidence of heterozygotes, three new amplifications and sequencing reactions were conducted as follows: only consistent double peaks were considered to be an indicator of a heterozygous site. The alignment and phasing of the entire dataset were performed using MUSCLE [29] and PHASE [30], respectively, and the haplotypes were unambiguously reconstructed. Due to the assumed maternal inheritance of the cpDNA with a low recombination rate, the *trn*D-*trn*T and *trn*H-*trn*K spacers were concatenated and will henceforth be jointly referred to as DTHK. To understand the genealogical relationships among the ACEP region samples, we applied the median joining method [31] implemented in PopART (http://popart.otago.ac.nz/index.shtml [32]), using default settings to consider multifurcations and/or reticulations in a phylogenetic network approach.

We next determined the haplotype frequencies of each sample and calculated the haplotype diversity (h), nucleotide diversity (π), and estimates of group pairwise Φ_ST_ values, considering the haplotype frequency using Arlequin 3.5 [33]. For further population-level analyses, we only considered groups with eight or more individuals per group (Table 1). The pairwise Φ_ST_ matrix was then dimensionally represented using multidimensional scaling (MDS) in R software [34]. The global values of G_ST_, Nei’s coefficient of multiple alleles gene differentiation analog to Wright’s F_ST_ [35,36], were inferred using DnaSp5.1 [37], considering gaps as the fifth state and haplotype data information [36]. Then, to compare the migration via pollen and seed, we estimated the pollen-to-seed migration ratio as (r = m_p_/m_s_ = {(1/G_STbipar_ − 1)(1 + F_IS_) − 2 (1/G_STmat_ − 1)}/(1/G_STmat_ − 1)) [38], given the global G_ST_ of each marker (G_STbipar_ for ITS and G_STmat_ for DTHK) and the previous average values of F_IS_ estimated for *A. germinans* (0.174) and *A. schaueriana* (0.242) using microsatellites [26]. By doing so, we assumed that the extent of inbreeding, F_IS_, is constant through time and also across the species populations.

**Table 1:** **Descriptive statistics for the** ***Avicennia*** **samples**

		**ITS**						**DTHK**					
**Geographic region**	**Statistics**	**n**	**N**_**subst**_	**π**	***h***	**(SD)**	**N**_**hap**_	**n**	**N**_**subst**_	**π**	***h***	**(SD)**	**N**_**hap**_
IWP	*A. alba*	1	0	0	0	(0)	1	1	0	0	0	(0)	1
ACEP	*A. bicolor*	6	0	0	0	(0)	1	6	0	7.091	0	(0)	1
IWP	*A. marina*	2	1	0.667	0.667	(0.204)	2	2	1	399.333	0.667	(0.204)	2
North Pacific	AgNPc	10	5	0.947	0.795	(0.065)	6	8	0	0	0.433	(0.138)	3
South Pacific	AgSPc	20	10	3.358	0.922	(0.022)	16	18	1	0.203	0.298	(0.093)	3
West Africa	AgAfr	4	1	0.429	0.429	(0.169)	2	1	0	0	0	(0)	1
North Atlantic	AgNAt	6	0	0	0	(0)	1	2	0	0	0	(0)	1
South Caribbean	AgSCr	7	1	2.901	0.264	(0.136)	2	1	0	0	0	(0)	1
Marajó, Brazil	AgMRJ	24	2	0.083	0.728	(0.041)	8	24	1	0.223	0.223	(0.072)	2
Pará*, Brazil	AgPAa	25	1	0.115	0.418	(0.086)	7	25	4	0.601	0.353	(0.083)	4
Pará, Brazil	AgPAb	16	5	0.71	0.698	(0.08)	7	16	9	1.427	0.389	(0.106)	5
Alcântara, Brazil	AgALC	21	1	0.418	0.519	(0.091)	8	21	8	2.499	0.4	(0.085)	3
Parnaíba, Brazil	AgPNB	24	1	0.284	0.301	(0.083)	4	24	0	0	0	(0)	1
Paracuru, Brazil	AgPRC	4	4	1.821	0.607	(0.164)	3	8	4	3.857	0.429	(0.169)	2
Natal, Brazil	AgNTL	2	0	0	0	(0)	1						
Tamandaré, Brazil	AgTMD	24	3	0.319	0.301	(0.083)	4	24	1	0.383	0.401	(0.072)	3
South Caribbean	AsSCr	1	0	0	0	(0)	1	1	0	0	1	(0.5)	2
Pará, Brazil	AsPAR	26	4	0.51	0.59	(0.069)	6	26	11	1.988	0.793	(0.05)	12
Alcântara, Brazil	AsALC	22	0	0	0.453	(0.085)	5	22	0	0	0	(0)	1
Paracuru, Brazil	AsPRC	16	0	0	0.669	(0.035)	3	16	0	0	0	(0)	1
Natal, Brazil	AsNTL	1	0	0	0	(0)	1	1	0	0	0	(0)	1
Vera Cruz, Brazil	AsVER	16	8	1.196	0.341	(0.105)	5	16	2	0.242	0.234	(0.095)	3
Guapimirim, Brazil	AsGPM	24	5	2.097	0.781	(0.045)	12	24	1	0.082	0.082	(0.053)	2
Ubatuba, Brazil	AsUBA	21	0	0	0.251	(0.078)	2	21	0	0	0	(0)	1
Cananéia, Brazil	AsCNN	23	0	0	0.125	(0.063)	2	23	0	0	0	(0)	1
Pontal do Paraná, Brazil	AsPPR	23	4	1.337	0.477	(0.087)	7	23	1	0.085	0.085	(0.055)	2
Florianópolis, Brazil	AsFLN	22	0	0	0	(0)	1	22	2	0.178	0.254	(0.085)	4

The species names and sample abbreviations are identical to those used in Figure 1 and Additional file 1. n, sample size; N_subst_, number of substitutions; π, nucleotide diversity; *h* (SD), haplotype diversity and (standard deviation of haplotype diversity); N_hap_, number of haplotypes.

To better understand the phylogeographic patterns of the observed genetic variation, and because the previously analyzed sequences were obtained from a few samples from each location [18,23], we arbitrarily grouped them into “geographic regions” according to previous studies [18,23,26] (see Additional file 1). Employing Arlequin 3.5 software [33], we studied the geographic distribution of the genetic diversity using a hierarchical analysis of molecular variance (AMOVA) [39] that considered different hypotheses for cpDNA and nDNA for each species. We created different *a priori* hypotheses regarding *A. germinans* and *A. schaueriana* based on several factors: a) the geographic influences of the American continent, b) the effects of contemporary near-surface marine currents on the genetic diversity of ACEP mangrove species [17,18,20,25,26,40], and c) the forest continuum of the Amazon Macrotidal Mangrove Coast (AMMC) [41], which includes samples from Pará and Maranhão States, (Figure 1 and Additional file 1). We also tested *a posteriori* groups regarding the genealogical analysis and the geographic distribution of haplotypes. The criteria for determining the best hypothesized arrangement were a significant departure from a random distribution and the maximum variance among groups (Φ_CT_). We used PERMUT software [42] to test whether different haplotypes that occurred within populations were more closely related than distinct haplotypes from different population by estimating and comparing the N_ST_, which considers both haplotype frequencies and their divergence, and G_ST_, which only considers haplotype frequencies, based on 10,000 random permutations.

We evaluated the demographic fluctuations using several summary statistics and considered the groups that best met the maximum significant Φ_CT_ criterion and the sample arrangements previously inferred using other genetic markers, such as microsatellites [17,18,23,26]. This approach of evaluating two distinct scenarios is justified by the differences between the sets of markers that were previously used to study the genetic diversity and the markers used in this study. We evaluated different neutrality tests: Tajima’s D [43] and Fu’s F_S_ [44] using Arlequin 3.5 [33] and D* and F* [45] computed with DnaSP 5.1 [37]. Assuming the loci to be selectively neutral, we justified the use of these statistics by their different statistical power and sensitivity to recombination [46]. We then considered Fu’s F_S_ [44] for the DTHK marker and Tajima’s D [43], D* and F* [45] for the ITS marker because, as expected, the latter presented more evidence of recombination than the former (data not shown). We then used Arlequin 3.5 to calculate the mismatch distribution of the observed number of differences between haplotype pairs to evaluate demographic expansions by analyzing the raggedness index [47]. These analyses of the distributions of pairwise differences were considered to be complementary evidence of demographic expansions when neutrality tests significantly departed from random distributions due to their conservativeness [48]. Regarding the ITS region, when only D* and F* [45] are significant, background selection is indicated as the likely mechanism underlying the polymorphism, and the opposite suggests population growth [44]. Significant negative values of Tajima’s D and Fu’s Fs are evidence of population growth, whereas a significant positive Tajima’s D is associated with population decline. Population expansion would also lead to a smooth mismatch distribution, and small raggedness values indicate a smooth mismatch distribution.

### Ethics statement

We obtained two licenses (Nos. 17159 and 17130) to collect the leaves and propagules of *A. germinans* and *A. schaueriana* from the Brazilian Institute of the Environment and Natural Renewable Resources - IBAMA (currently Chico Mendes Institute for Biodiversity Conservation - ICMBio). We confirm that *A. germinans* and *A. schaueriana* are not endangered or protected species.

## Results

**Figure 2:**
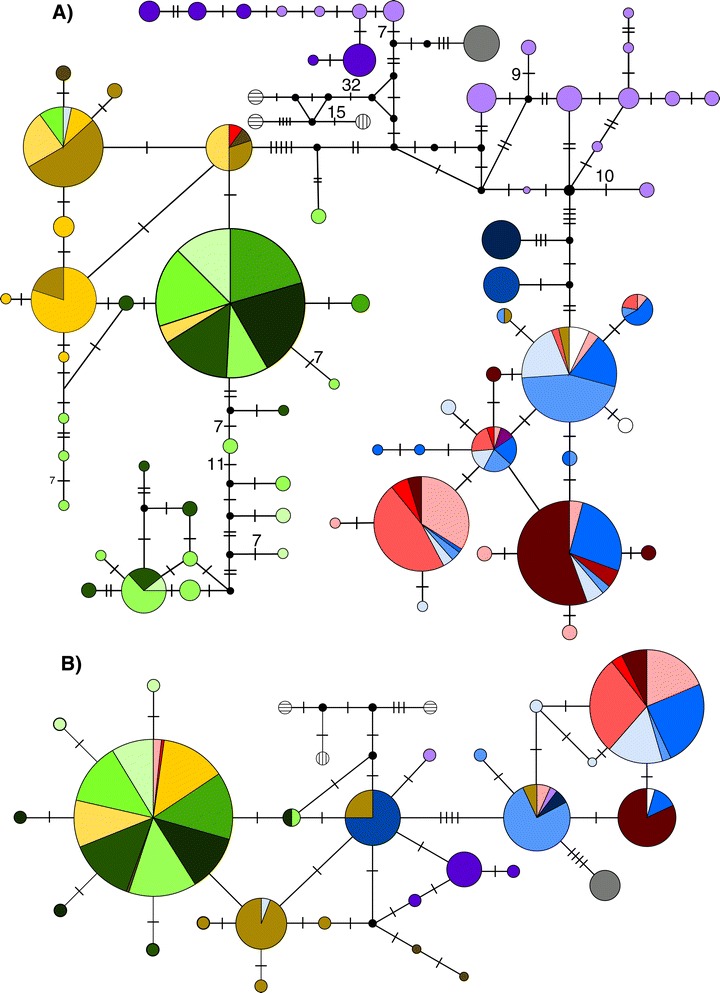
**Median joining networks of cpDNA and nDNA of** ***Avicennia*** **species** ***.*** The haplotype networks of the **A)** ITS and **B)** DTHK markers in *Avicennia* species from the Western, *A. bicolor, A. germinans* and *A. schaueriana*, and Eastern Hemispheres, *A. marina* and *A. alba*. Each line in the network refers to a single-nucleotide mutation, the double bars combined with numbers indicate the numbers of mutations between haplotypes, and the black dots indicate missing haplotypes in the samples. The circles denote unique haplotypes and are proportional to the number of sequences, with colors representing the samples according to Figure 1 and Additional file 1. *A. marina* and *A. alba*, species from the Eastern Hemisphere, are denoted as circles with vertical and horizontal lines, respectively

To evaluate the distribution of the genetic diversity of the three *Avicennia* species on the scale of the entire Western Hemisphere, we obtained samples of *A. germinans* and *A. schaueriana* from northeastern and southern South America and studied them together with previously evaluated samples using cpDNA and nDNA markers [18,23]. The total number of individuals per sample and the descriptive statistics regarding the genetic diversity are shown in Table 1. The numbers of polymorphic sites we observed were 91 and 129, totaling 28 and 72 haplotypes for the DTHK and ITS loci, respectively; the haplotype (*h*) and nucleotide (π) diversities (Table 1) varied substantially among populations. As expected, each species had unique haplotypes for each marker, but we observed shared haplotypes between individuals identified as *A. germinans* and *A. schaueriana* along the northeastern coast of South America (Figure 2). One of these shared ITS and DTHK haplotypes was also observed in the African *A. germinans* samples (Figure 2).

**Table 2:** **Analysis of molecular variance for five different grouping models for** ***A. germinans*** **and** ***A. schaueriana***

***A. germinans***												
			**DTHK**					**ITS**				
**Hypothesis**		**Hypothesized grouping**	**Φ**_**SC**_	**Φ**_**ST**_	**Φ**_**GT**_	**% Among groups**	**P Φ**_**GT**_	**Φ**_**SC**_	**Φ**_**ST**_	**Φ**_**GT**_	**% Among groups**	**P Φ**_**GT**_
Ag1	*a priori*	[Atlantic][Pacific]	0.631	0.222	−1.112	−111.210	0.17822 ± 0.00343	0.438	−0.115	−0.983	−98.340	0.10891 ± 0.00318
Ag2	*a priori*	[Atlantic][AgNPc][AgAgSPc]	0.668	0.385	−0.854	−85.440	0.85297 ± 0.00321	0.668	0.385	−0.854	−85.440	0.85297 ± 0.00321
Ag3	*a priori*	[North Brazil][South Brazil][Pacific]	0.722	0.313	−1.470	−147.010	0.83010 ± 0.00390	0.534	−0.331	−1.853	−185.350	0.52743 ± 0.00479
Ag4	*a priori*	[AgNAt][AgSAt][AgSPc][AgNPc]	0.837	0.802	−0.218	−21.780	0.35881 ± 0.00483	0.786	0.775	−0.052	−5.240	0.27693 ± 0.00469
Ag5	*a priori*	[AgSPc][AgNPc][AMMC][AgPNB][AgTMD]	−1.697	1.162	1.060	106.020	0.25505 ± 0.00461	2.015	1.082	0.919	91.920	0.15188 ± 0.00399
Ag6	*a posteriori*	[AgSPc][AgNPc][AgPAa][AgTMD]	−0.107	1.117	1.106	110.560	0.05733 ± 0.00228	−2.430	1.149	1.043	104.340	0.32436 ± 0.00428
		[AgPAb + AgTMD + AgPNB]										
Ag7	*a posteriori*	[AgSPc][AgNPc][AMMC][AgPAa]	−0.019	0.978	0.978	97.820	0.30198 ± 0.00477	−0.211	0.949	0.958	95.810	0.38851 ± 0.00487
		[AgTMD][AgPNB]										
Ag8	*a posteriori*	[AgSPc][AgNPc][AgPAa + AgTMD]	0.262	0.830	0.770	77.010	0.03465 ± 0.00186	7.792	1.028	0.996	99.590	0.77861 ± 0.00409
		[AgPab + AgMRJ + AgALC][AgPNB]										
***A. schaueriana***												
			**DTHK**					**ITS**				
**Hypothesis**		**Hypothesized grouping**	**Φ**_**SC**_	**Φ**_**ST**_	**Φ**_**GT**_	**% Among groups**	**P Φ**_**GT**_	**Φ**_**SC**_	**Φ**_**ST**_	**Φ**_**GT**_	**% Among groups**	**P Φ**_**GT**_
As1	*a priori*	[North Brazil][South Brazil]	0.491	−1.432	−3.775	−377.530	0.70446 ± 0.00405	0.255	4.543	5.756	575.630	0.99000 ± 0.00098
As2	*a priori*	[AMMC][AsPRC][South Brazil]	0.532	−1.321	−3.958	−395.810	0.67614 ± 0.00471	0.291	3.318	4.270	426.970	0.96515 ± 0.00183
As3	*a posteriori*	[AsPAR][AsALC + AsPRC][South Brazil]	−0.044	1.685	1.656	165.650	0.90881 ± 0.00277	0.333	3.617	4.922	492.150	0.97257 ± 0.00164
As4	*a posteriori*	[AsPAR][AsALC + AsPRC+ AsGPM+ AsPPR + AsVER + AsUBA + AsFLN + AsCNN]	−0.030	5.706	5.569	556.910	1.000	0.442	−0.056	−0.893	−89.280	1.00000 + −0.00000
As5	*a posteriori*	[AsPAR][AsALC + AsPRC][AsGPM + AsPPR]	−0.145	1.080	1.070	106.990	0.96525 ± 0.00180	0.583	1.193	1.462	146.150	0.99436 ± 0.00081
		[AsVER + AsUBA + AsFLN + AsCNN]										
As6	*a posteriori*	[AsPAR]{ AsALC + AsPRC][AsVER]	−0.021	0.458	0.470	46.970	0.01406 ± 0.00122	3.619	1.065	0.975	97.510	0.01436 ± 0.00109
		[AsGPM + AsPPR + AsUBA + AsCNN][AsFLN]										
As7	*a posteriori*	[AsPAR][AsALC + AsPRC][AsVER][AsGPM]	0.018	0.869	0.867	86.650	0.85396 ± 0.00306	−0.168	0.858	0.878	87.810	0.81059 ± 0.00375
		[AsUBA + AsCNN][AsPPR][AsFLN]										
As8	*a priori*	[AsPAR][AsALC][AsPRC][AsGPM]	−0.016	0.418	0.427	42.720	0.53287 ± 0.00505	0.001	0.882	0.882	88.220	0.89436 ± 0.00248
		[AsPPR + AsVER + AsUBA][AsCNN + AsFLN]										
As9	*a priori*	[AsPAR][AsALC][AsPRC][AsGPM][AsVER]	0.001	0.919	0.919	91.870	0.33327 ± 0.00491	−0.012	0.911	0.912	91.150	0.35238 ± 0.00448
		[AsUBA][AsPPR + AsCNN + AsFLN]										

Analysis of molecular variance for different grouping models based on previous hypotheses regarding the genetic structure based on microsatellite markers on the current distribution of mangrove forest (*a priori* hypotheses) and on the genealogical relationships of the haplotypes (*a posteriori* models). The acronyms refer to the geographic regions where samples were obtained and are identical to those used in Additional file 1. The samples labeled “North Brazil” were obtained from the states of Pará, Maranhão, Piauí and Ceará, and the samples labeled “South Brazil” were the remaining samples from the Brazilian coastline regions. AMMC designates samples from the Amazon Macrotidal Mangrove Coast from Pará and Maranhão States.

### Genealogical relationships

The median-joining haplotype network of each marker indicated a deep divergence between the IWP and ACEP species and an intricate relationship among the samples of the latter (Figure 2). At the species level, as expected, the haplotypes were mostly congruent with each taxon, indicating complete lineage sorting for both the ITS and the DTHK. However, the geographic distribution of the haplotypes was slightly different when each of these markers was considered. Regarding the nDNA sequences (Figure 2A), there was a strong relationship between the geographic origin and haplotype for some samples, such as the Pacific, southern Caribbean and North Atlantic samples of *A. germinans.* However, when mainly *A. germinans* and *A. schaueriana* samples from the Brazilian coast were considered, there was no obvious pattern of genetic structure due to haplotype sharing among samples from different geographic origins. A highly divergent group composed of the AsPPR, AsGPM and AsVER samples was also observed, supporting the ITS phylogenetic tree (Figure 2A). Given the cpDNA sequences (Figure 2B), the geographic structure of the genetic diversity was similar to that observed for the nDNA marker: *A. germinans* samples from West Africa, the North Atlantic and the southern Caribbean composed a distinct group, and the Pacific haplotypes composed another clear cluster. As was observed for the ITS marker (Figure 2A), the Brazilian samples of *A. germinans* presented a more complex phylogeographic pattern. In the *A. schaueriana* samples, there was a dominant haplotype that was shared by most of the individuals, whereas the AsPAR samples presented a group of closely related haplotypes.

Regardless of the marker considered, we found evidence of ‘star-like’ genealogies [49], where sampled lineages experienced independent evolution since their most recent common ancestor, which may be considered as preliminary signals of recent demographic expansions [50,51]. Moreover, the haplotype network constructed for both markers demonstrated that some individuals identified as *A. germinans* presented haplotypes that are in much higher frequency in *A. schaueriana* individuals and that this observation was reciprocal.

### Population-level analyses of *A. germinans* and *A. schaueriana*

Population differentiation analyses indicated that there was intraspecific genetic divergence between the evaluated samples of *A. germinans* and *A. schaueriana* (global G_ST_ values [36] of 0.568 and 0.340 for the former, and 0.397 and 0.386 for the latter, for the DTHK and ITS markers, respectively). The differences between inferences of G_ST_ by means of these markers indicated that *A. germinans* had a pollen-to-seed ratio of r = 0.996, whereas the pollen-to-seed ratio of *A. schaueriana* was negative or practically zero (r = −0.699). This difference suggests that the gene flow of *A. germinans* through its propagules was similar to the gene flow by pollen; however, in *A. schaueriana*, the movement of genes by seeds was one to two times higher than that by pollen.

**Figure 3:**
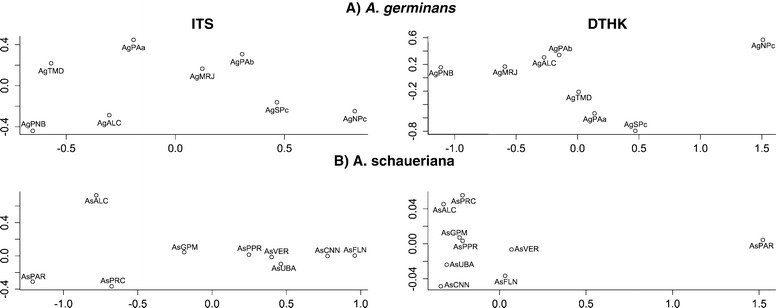
**Multi-dimensional scaling (MDS) of pairwise Φ**_**ST**_ **among** ***Avicennia*** **samples.** MDS of pairwise Φ_ST_ among samples of **A)** *A. germinans* and **B)** *A. schaueriana* based on the ITS and DTHK markers (see Additional file 2). The sample abbreviations are the same as those used in Figure 1 and Additional file 1.

The G_ST_ values indicated that there was substantial genetic structure, which is more readily observed when one considers the pairwise Φ_ST_ values for each species, which were mostly significant, except for the cpDNA marker and the *A. schaueriana* samples (see Additional file 2). The overall organization of the genetic diversity was complex, as shown by the graphical representation of the MDS analyses (Figure 3), which resulted in relatively reliable models; the lowest measure of the goodness of fit when considering the two dimensions was 0.7893.

To better understand this intricate pattern of genetic structure, we explicitly tested for different geographic grouping hypotheses (Table 2). Despite previous studies that found evidence of genetic structure in three mangrove species (*A. germinans, A. schaueriana* and *R. mangle*) from the ACEP region [17,20,26], the AMOVA results considering the cpDNA and nDNA markers of both *A. germinans* and *A. schaueriana* indicated that *a posteriori* hypotheses provided better explanations for the observed molecular variation. The *a priori* models considered different combinations of the expected effects of the AMMC, the American continent and/or prominent surface marine currents on the genetic diversity. The hypothesized groupings of Ag1, Ag4, As1, As8 and As9 were based on previous studies that used molecular markers to evaluate the genetic variation [17,18,20,23,52]. In general, these hypotheses performed worse than our *a posteriori* models because the Φ_CT_ values were not significantly different from random distributions, and due to the highly negative values of among-group variance, they failed to reasonably explain the genetic diversity (Table 2). For *A. germinans,* no hypothesis was consistently supported by the AMOVA outcomes; coupled with the high and significant pairwise Φ_ST_ values. This finding indicates that the genetic variation is likely to be organized among samples with relatively limited gene flow among them. Conversely, for *A. schaueriana,* model As6 was consistently supported by both the DTHK and ITS markers despite the extremely low variation among groups (Φ_ST_ = 0.46969) when the former was analyzed (Table 2).

Considering both the genetic structure identified herein (in model As6 and when considering each sample separately for *A. germinans*) and the *a priori* scenarios, namely models Ag1 and As1, based on previous studies [17,18,20,25,26,40], we observed different indications of recent demographic expansions in both species from both the *a priori* and *a posteriori* hypotheses. When *A. germinans* model Ag1 was considered, we found signs of expansion in the AgNPc and AgNAtl groupings, whereas AgSPc showed indications of background selection. When each population was considered, only AgALC, AgPNB and AgTMD did not present signs of demographic changes (Table 3). These results are only supported by the ITS marker, whereas both the nDNA and cpDNA loci suggest that recent population growth has also occurred in *A. schaueriana.* Regarding both the hypothesized models, there were signs of expansion in every group except for AsFLN (Table 3).

Regarding the PERMUT analysis, we observed that for *A. germinans*, the provided ITS and DTHK markers pointed to a significant (P < 0.05) difference between the mean values of G_ST_ (0.274 and 0.528, respectively) and N_ST_ (0.559 and 0.733, respectively). In contrast, for *A. schaueriana* , the differences between these indexes were not significant (P > 0.05): for ITS and DTHK markers, the mean values of G_ST_ were 0.384 and 0.442, respectively, whereas the mean values for N_ST_ were 0.302 and 0.400, respectively.

## Discussion

### The presence of interspecific hybridization in *Avicennia* species at the Western Hemisphere

By means of both haplotype networks, we observed that the three *Avicennia* species from the ACEP region may be considered three different evolutionary lineages independent from the lineage composed of *A. alba* and *A. marina,* which are IWP species (Figure 2). However, the isolation of these species is not absolute. Although there is no evidence of ongoing hybridization between *A. bicolor* and *A. germinans,* an ancient introgression between them has already been reported [23], and evidence for this historical contact between these species (the incongruent phylogenetic relationship between the cpDNA and the nDNA) was also observed in this work with the inclusion of more samples of *A. germinans* and *A. schaueriana* from the southeastern coast of South America (Figure 2).

On the other side of the American continent, even more interestingly, we found new evidence of current hybridization between *A. germinans* and *A. schaueriana.* Using microsatellites, we have previously observed that these two species may interbreed, and, furthermore, that this hybridization is asymmetric because only individuals identified as *A. germinans* presented signals of interspecific breeding (evidence of F1 individuals and trees originated from backcrosses between F1 hybrids and *A. germinans* within the zone of sympatry for this species and *A. schaueriana.*) [26]. Herein, we find additional evidence of this hybridization, but the new data do not support this asymmetry. Using DTHK and ITS haplotype sharing, we found additional evidence of interbreeding between *A. schaueriana* and *A. germinans* from several locations within the zone of sympatry for these species, indicating that this biological process may be more common than previously believed. We favor hybridization/introgression rather than an ancestral polymorphism as the most likely mechanism generating this haplotype sharing due to the positions in the phylogenetic networks and the relatively high frequencies of the shared haplotypes. The branches where these haplotypes occurred, however, are not more related to the IWP species, as would be expected for this biological process (Figure 2), and two of the four haplotypes shared by these species were rare (less than 3%), whereas ancestral haplotypes are presumed to be more frequent. Moreover, because there are individuals that were identified as either *A. germinans* and *A. schaueriana,* while sharing reciprocal haplotypes, cpDNA and nDNA data no longer support the asymmetrical hybridization between these species, indicating that gene flow may indeed occur bidirectionally.

These observations suggest that introgressive hybridization is a more widespread process on both coasts of the American continent for *Avicennia* than previously believed, adding a relevant report to the large list of examples of hybridization in mangrove species. Based on the morphological and molecular data, interspecific gene flow has been described for the genera *Rhizophora, Bruguiera* (Rhizophoraceae)*, Sonneratia* (Lythraceae)*, Lumnitzera* (Combretaceae) and *Avicennia* [1,23,52-57]. Although we can speculate on the mechanisms that maintain the widespread breeding between related mangrove species and on the evolutionary consequences of this process, we prefer to encourage further genetic and ecological studies regarding these intriguing questions.

### Geographic distribution of intraspecific genetic diversity

At the species level, *A. bicolor, A. germinans* and *A. schaueriana* presented clear genetic differentiation despite the evidence for introgression previously discussed here and elsewhere [23]. Conversely, at the intraspecific level, the organization of the genetic variation in *A. germinans* and *A. schaueriana* was not obvious.

The Pacific and Atlantic samples of *A. germinans* clearly clustered into different groups. The samples from the west coast of Central America were mostly phylogenetically separate from the remaining haplotypes, which can be readily visualized in ITS and DTHK haplotype networks (Figure 2). However, for the chloroplast marker (Figure 2A), there was haplotype sharing between samples from the Pacific coast of Mexico and from the Atlantic coast of the American continent. This distribution is most likely explained by intraspecific ancestral polymorphism because of the low frequency of the shared haplotype (Figure 2B) and because the Isthmus of Panama is a strong barrier to pollen flow for this insect-pollinated species [25]. Another explanation for this sharing is that past sea-level fluctuations may have facilitated pollen gene flow, as has been proposed for *Hibiscus pernambucensis* Arruda (Malvaceae) [58], whose pollination is also based on insects. In total, despite the evident Pacific-Atlantic differentiation [18,23,25,40], the evolutionary scenario in the Atlantic basin, where our sampling size was larger, is complex.

Individuals from both sides of the Atlantic Ocean shared ITS and DTHK haplotypes, and those haplotypes that were different were phylogenetically closely related (Figures 2); this observation has already been reported using the same set of markers [23] and PCR-restriction fragment length polymorphism coupled with chloroplast microsatellites [18]. The new results that we present in this study indicate that the LDD between Africa and America is a more common process than previously thought, corroborating the previous rejection of a vicariant process to explain the widespread distribution of *A. germinans* [18]. Despite the drawbacks of the use of ITS for genus and species level evolutionary studies such as homoplasy, loci duplication and contamination due to its universality [59,60], we argue that the ITS haplotype sharing we observed is valid but not stand-alone evidence of *A. germinans* transatlantic seed dispersal.

This finding supports and extends the role of transatlantic dispersal as a relevant evolutionary process for the mangrove species *A. germinans* [18] and *R. mangle* [20], whose propagules may float, survive and even produce roots after long periods in fresh and salt water [61]. Moreover, LDD across the Atlantic Ocean has also been reported for *Hibiscus* L., a sea-dispersed plant, [58,62] and even for a species whose seeds have no adaptations for water dispersal [63]. This movement between the west coast of Africa and the east coast of South America is most likely driven by the high surface velocity of the westward southern South Equatorial Current (SEC) [64].

Interestingly, despite the high longevity of *A. germinans* propagules in salt and fresh water and their high buoyancy (*A. germinans* propagules always float, even when rotten) [61], LDD is likely not a relatively frequent process for this species. This mechanism is likely to be rare enough that there is no generalized homogenization of the species genetic diversity [26]. We have previously reported that there was genetic structure on different geographic scales along the Brazilian coast, with significant genetic differentiation between samples separated by distances from thousands of kilometers to hundreds of meters, regarding microsatellite analyses in both *A. germinans* and *A. schaueriana* [26]. Moreover, limitations of gene flow, even within estuaries, have been reported in Central America for *A. germinans* and *R. mangle* [25]*.* The observation of both long-distance dispersal and limited dispersal was also observed for mangrove species from the Eastern Hemisphere, e.g., *Rhizophora* [65,66], *Ceriops* (Rhizophoraceae) [67-69] and *Kandelia* (Rhizophoraceae) [70-72] species. Whether this pattern of limited and, intriguingly, long-distance dispersal is a general feature of mangrove biology remains to be tested.

An evaluation of DTHK and ITS markers supports these results; in this study, we observed that, although there was considerable haplotype sharing among the *A. germinans* samples, there was also generally substantial and significant genetic differentiation, as measured by global G_ST_, its comparison with N_ST_ and the pairwise Φ_ST_ (see Additional file 2) with a complex pattern in the MDS plot (Figure 3A). The most robust hypothesis of genetic organization by the hierarchical AMOVA corroborates these results because the most reliable hypothesis was generated by considering each of the samples separately (Table 2), supporting the pattern that explains small geographic scale structure using microsatellites [26]. This result indicates that the historical and current propagule dispersal of *A. germinans* is limited and usually occurs locally; for example, dispersal may occur in a forest continuum, such as the AMMC, or within a single estuary, such as in Central America [25].

In *A. schaueriana,* there was also a complex relationship between the genealogical inferences and the geographic distribution of haplotypes. Many of the haplotypes were shared by different and geographically distant samples (Figure 2), and we similarly observed a high level of genetic structure, as revealed by global G_ST_ measures. Despite the notable differences between the DTHK and ITS results regarding the pairwise Φ_ST_ (see Additional file 2), which are easily observed in the MDS plot (Figure 3B), one *a posteriori* grouping was consistently supported by both markers when we considered the hierarchical AMOVA outcomes (Table 2). The As6 model differed slightly from the models that examined small-scale genetic structure using microsatellites [26] and the tested *a priori* groupings (models As8 and As9 – Table 2), and it could explain the nonsignificant difference between N_ST_ and G_ST_ from the PERMUT analysis for *A. schaueriana.* The samples from this species are probably not from independent populations, in contrast to what we observed for *A. germinans*. The relatively low variance among groups (46.97%) for the DTHK marker may be explained by the remarkable genetic diversity of this marker that was observed in AsPAR compared to other *A. schaueriana* samples (Table 2); this diversity produced a large proportion of the molecular variability within the samples (54.16%).

As a whole, the most likely groupings hypothesized herein disagree with the most feasible evolutionary scenarios inferred by means of microsatellite data in *A. germinans* and *A. schaueriana* [26] and other sea-dispersed plants, including *R. mangle* [17,20] and *H. pernambucensis* [58]. For all of these species, a similar pattern of genetic structure was observed, with a clear distinction between the samples that were collected from sites north and south of the northeastern extremity of Brazil (Figure 1). Models Ag4 and As1 rely on this pattern of genetic structure and poorly explained the molecular variation we observed (Table 2). This finding indicates that there are, most likely, different historical and ongoing processes influencing the genetic diversity of these *Avicennia* species due to the differences in the mutation rates between these sequence-based markers and microsatellites [73-75].

### Historical and ecological processes shape the genetic diversity of *A. germinans* and *A. schaueriana*

The line of reasoning mentioned above supports the hypothesis that *Avicennia* has been affected by historical demographic changes in the ACEP region (more precisely, in the Pacific basin of Central America [18,19] and more broadly in the Eastern Hemisphere [76]). During glacial periods, high-latitude edge populations would have become extinct and would subsequently have been recolonized by individuals from core regions near the Equator [18,76]. The *A. germinans* and *A. schaueriana* samples in this study did not indicate a higher genetic diversity poleward by means of either the DTHK or ITS markers (Table 1) or by microsatellites [26]. However, the disagreement between the most likely scenarios considering high (Ag4 and As1) and low mutation rates indicates that different processes have shaped and continue to influence the species’ genetic diversity. We argue that a similar process most likely occurred along the Atlantic coast of South America.

**Table 3:** **Neutrality tests for** ***A. germinans*** **and** ***A. schaueriana***

**A) ** ***A. germinans***												
**ITS**	**model Ag1**				***A. germinans*** ** populations**							
**Statistics**	**AgNPc**	**AgSPc**	**AgNBr**	**AgTMD**	**AgNPc**	**AgSPc**	**AgMRJ**	**AgPAa**	**AgPAb**	**AgALC**	**AgPNB**	**AgTMD**
Tajima’s D	*−3.350*	−1.606	*−2.412*	−1.478	*−3.350*	−1.606	*−2.408*	*−2.173*	*−2.988*	−1.848	−1.210	−1.478
D*	*1.406*	*1.506*	0.659	1.008	*1.406*	*1.506*	*1.654*	−0.829	1.368	1.188	0.895	1.008
F*	1.035	*1.762*	−0.390	0.491	1.035	*1.762*	0.656	−1.067	0.536	0.796	0.562	0.491
Raggedness index	0.728	*0.166*	*0.205*	0.243	0.728	*0.166*	0.705	0.605	0.167	0.201	0.267	0.243
DTHK												
FS	3.4*10^38^	−1.502	−1.984	−0.183	3.4*10^38^	−1.502	0.468	−0.402	−0.513	5.718	0	−0.183
Raggedness index	0	0.393	0.306	0.201	0	0.393	0.355	0.658	0.165	0.505	0	0.201
**B) ** ***A. schaueriana***												
**ITS**	**model As1**		**model As6**									
**Statistics**	**AsNBr**	**AsSBr**	**PAR**	**ALC_PRC**	**VER**	**GPM_PPR_UBA_CNN**	**FLN**					
Tajima’s D	*−2.721*	*−2.398*	*−2.804*	*−2.233*	*−3.094*	*−2.345*	0					
D*	0.543	0.477	1.315	−0.990	0.755	*1.751*	0					
F*	−0.264	0.511	0.643	−0.650	0.362	*1.839*	0					
Raggedness index	0.780	0.534	0.498	0	0	0.467	0					
DTHK												
FS	−8.218	*−11.061*	*−3.382*	0	*−3.642*	−3.637	−1.250					
Raggedness index	0.253	0.680	0.043	0	0.339	0.835	0.459					

Results of tests for neutrality and population expansion given two different evolutionary scenarios for A) *A. germinans* and B) *A. schaueriana* based on microsatellite, cpDNA and nDNA markers. The values of Tajima’s D (Tajima, 1989); Fu and Li’s D* and F*(Fu & Li, 1993); the raggedness index (Rogers & Harpending, 1992); and Fu’s FS (Fu, 1997) are shown. The values in italics indicate P < 0.02 for FS and P < 0.05 for the remaining statistics.

After extinction events occurred due to Quaternary environmental changes, populations would have become more isolated [77]. This disjoint distribution, coupled with the limited gene flow caused by relatively restricted pollen and propagule dispersal, would have enabled the evolution of distinct independent lineages that could later expand their geographic distribution after the glaciation. This evolutionary scenario explains the shared haplotypes between our studied samples and the genealogical relationships of the ITS marker (Figure 2A), which were observed in samples from three sites separated by hundreds of kilometers. This scenario is also consistent with the partial incongruence between the sequence-based and microsatellite genetic structures. To further test this hypothesis, we studied the eventual demographic expansion signals. If this evolutionary history is consistent, we would expect to observe significant evidence of demographic expansion across the inferred populations.

For both species, we tested whether the groupings that yielded the most likely genetic structure pattern with regard to microsatellite and sequence-based marker results (regarding models As1 and As6 for *A. schaueriana* and model Ag1 and considering each sample separately for *A. germinans*) presented signs of recent demographic change. For both species, in aggregate, we found signs of population growth for different evolutionary scenarios across the samples (Table 3). Contrary to our expectations, we found no signs of demographic expansion in the samples from the Pacific basin of southern Central America, which was presumably a refugium during the last glaciation [18,19]; instead, we observed indications of background selection in these samples. In the South American Atlantic basin, we found that *A. germinans* and *A. schaueriana* most likely responded differently to the post-glacial period. Whereas *A. germinans* only showed evidence of population growth on the northern coast of Brazil (model Ag1, and in AgMRJ, AgPAa and AgPAb when each sample was evaluated), there were consistent indications that recent demographic expansion occurred along the entire *A. schaueriana* distribution regardless of the model that was assumed (Table 3).

The differences between the patterns of recent population growth explains the current geographic distribution of these species along the Atlantic Coast of South America (Figure 1) because we found more substantial signs of demographic expansion (with evidence from both DTHK and ITS) in a broader geographic extension for *A. schaueriana* than for *A. germinans*. We argue that because the southern limit of the *A. schaueriana* distribution presents temperatures within the range of variation of *A. germinans* [4] and because this climatic factor is regarded as a major driver that influences mangrove latitudinal limits [3], additional major traits must influence the distributions of these species in South America. This pattern of geographic distribution may have been originated by an ecological difference between these species; the unequal historical effectiveness of relative gene flow may have resulted from pollen and propagule dispersal. *A. germinans* pollen and propagules contributed similarly to the gene flow; however, in *A. schaueriana,* we observed that gene flow via sea-water dispersed propagules was one to two times higher than gene flow via pollen along the Brazilian coast. This difference may imply a more efficient dispersal that could have enabled *A. schaueriana* to colonize the southern and southeastern coast of Brazil. These inferences of past pollen to seed gene flow are similar to those observed for *A. germinans* from the Pacific and Atlantic basins of Panama, with r = −0.64 [25]; however, because different sets of molecular markers were used, direct comparisons between these studies are not possible.

## Conclusions

The *Avicennia* species from the ACEP region presented genetic structuring at different levels of organization. *A. bicolor, A. germinans* and *A. schaueriana* are distinct evolutionary lineages whose boundaries are not complete because there is evidence for past [23] and ongoing introgressive hybridization processes on the American continent. Given the intraspecific level, in addition to finding new evidence of transatlantic LDD of *A. germinans* that may contribute to its widespread distribution within the South American Atlantic basin, we observed partially discordant molecular variation patterns between high (microsatellites - [26]) and low (DTHK and ITS – present work) mutation rate markers for both *A. germinans* and *A. schaueriana*. We argue that this discordance is likely due to a recent demographic expansion of both species, whose patterns diverge between these species. This disagreement, coupled with a larger proportion of gene flow brought by propagules rather than pollen in *A. schaueriana* but not in *A. germinans,* explains the current distribution of these species in South America.

In addition to these retrospective conclusions, the novel details that our findings revealed about the evolutionary history of the ACEP region *Avicennia* species can also provide valuable information about the responses of these plants to current global climate change. For example, despite their close phylogenetic relationship, *A. germinans* and *A. schaueriana* have responded differently since the last glaciation, and it is, thus, likely that their distinct ecological features may also influence their future in the face of the currently changing world. Considering this information about the past, our current endeavor is to understand potential impacts of the current climate changes on the neutral genetic variation of *A. germinans* and *A. schaueriana.*

## Additional files

Additional file 1:
***Avicennia*** **samples analyzed in this study.** Taxa, collection codes with sample sizes within parentheses for samples we collected, locations (degree decimals and geopolitical units) and geographic regions (with abbreviations in parentheses) of the samples evaluated using ITS (nDNA), *trn*D-*trn*T and *trn*H (cpDNA) markers. Additional file 2:
**Intraspecific pairwise genetic structure for** ***A. germinans*** **and** ***A. schaueriana.*** Pairwise Φ_ST_ between samples of A) *A. germinans* and B) *A. schaueriana.* The values below the diagonal were obtained using the ITS marker, and the values above the diagonal were obtained using the DTHK marker. The bold and underlined numbers indicate the nonsignificant pairwise Φ_ST_ values after 10,000 bootstraps.
