# Supplementary material for: Species distribution and introgressive hybridization of two Avicennia species from the Western Hemisphere unveiled by phylogeographic patterns
Source: BMC Evol Biol. 2015 Apr 10;15:61. doi: 10.1186/s12862-015-0343-z (PMC4394560; doi:10.1186/s12862-015-0343-z)
Supplement: Additional file 1: — Avicennia samples analyzed in this study. Taxa, collection codes with sample sizes within parentheses for samples we collected, locations (degree decimals and geopolitical units) and geographic regions (with abbreviations in parentheses) of the samples evaluated using ITS (nDNA), trnD-trnT and trnH (cpDNA) markers. [file 12862_2015_343_MOESM1_ESM.pdf]

Additional files

Additional file 1. *Avicennia* samples analyzed in this study.

Taxon	collection code	ITS	*trn*D-*trn*T	*trn*H	longitude	latitude	Locality	State	Country	Geographic region
A. marina (Forssk.) Vierh.										
A. marina	RSD-MaPe1	EF540978	EF540944	EU352163	-	-	Perth		Australia	
A. marina	RSD-MaMgI	DQ469861	EF540943	EU352164	-	-			Madagascar	
A. alba Blume										
A. alba	RSD-alb1	EF540977	EF540951	EU352162	-	-			Indonesia	
A. bicolor Standl.										
A. bicolor	RSD-Pb1	EF540988	EF540948	EU352159	10.566	-85.666	Playa Panama		Costa Rica	
A. bicolor	RSD-Pb10	EU352149	EU352149	EU352161	10.566	-85.666	Playa Panama		Costa Rica	
A. bicolor	RSD-Tm4	EF540989	EF540949	EU352167	9.85	-84.683	Tivives		Costa Rica	
A. bicolor	RSD-Tb1	EF540987	EF540950	EU352160	9.85	-84.683	Tivives		Costa Rica	
A. bicolor	RSD-Abt1	EU352150	EU352153	EU443251	15.933	-93.8	Tonalá	Chiapas	Mexico	
A. bicolor	RSD-Abt9	EU352151	EU352152	EU352158	15.933	-93.8	Tonalá	Chiapas	Mexico	
A. germinans (L.) L.										
A. germinans	RSD-8-15	DQ469844	EF540954	EU352182	8.4	-81.583	Chiriqui		Panama	South Pacific (AgSPc)
A. germinans	RSD-8-11	DQ469845	EF540955	EU352183	8.4	-81.583	Chiriqui		Panama	South Pacific (AgSPc)
A. germinans	RSD-8-6	DQ469846	EF540956	EU352184	8.4	-81.583	Chiriqui		Panama	South Pacific (AgSPc)
A. germinans	RSD-6-1	DQ469847	EF540957	EU352175	8.833	-79.566	Aguadulce		Panama	South Pacific (AgSPc)
A. germinans	RSD-5-9	DQ469849	EF540946	EU352176	8.833	-79.566	Aguadulce		Panama	South Pacific (AgSPc)
A. germinans	RSD-2-15	EF540984	EF540958	EU352177	8.645	-79.703	Punta chame		Panama	South Pacific (AgSPc)
A. germinans	RSD-TL11	EF540982	EF540959	EU352171	9.85	-84.683	Tivives		Costa Rica	South Pacific (AgSPc)
A. germinans	RSD-Tm17	EF540985	EF540960	EU352174	9.85	-84.683	Tivives		Costa Rica	South Pacific (AgSPc)
A. germinans	RSD-Tm6	EF540981	EF540961	EU352173	9.85	-84.683	Tivives		Costa Rica	South Pacific (AgSPc)
A. germinans	RSD-Pm8	EF540980	EF540972	EU352168	10.566	-85.666	Playa Panama		Costa Rica	South Pacific (AgSPc)

A. germinans	RSD-Pge8	EF540979	EF540973	EU352172	10.566	-85.666	Playa Panama		Costa Rica	South Pacific (AgSPc)
A. germinans	6_10	DQ469848			8.833	-79.566	Aguadulce		Panama	South Pacific (AgSPc)
A. germinans	11_5	DQ469843			10.066	-84.966	Punta Morales		Costa Rica	South Pacific (AgSPc)
A. germinans	RSD-14-3	DQ469837	EF540963	EU352186	10.3	-85.25	Tempisque		Costa Rica	South Pacific (AgSPc)
A. germinans	RSD-14-2	DQ469838	EF540962	EU352187	10.3	-85.25	Tempisque		Costa Rica	South Pacific (AgSPc)
A. germinans	RSD-13-8	DQ469840	EF540964	EU352188	10.3	-85.25	Tempisque		Costa Rica	South Pacific (AgSPc)
A. germinans	RSD-13-6	DQ469839	EF540965	EU352189	10.3	-85.25	Tempisque		Costa Rica	South Pacific (AgSPc)
A. germinans	RSD-13-4	DQ469841	EF540966	EU352190	10.3	-85.25	Tempisque		Costa Rica	South Pacific (AgSPc)
A. germinans	RSD-11-9	DQ469842	EF540953	EU352191	10.066	-84.966	Punta Morales		Costa Rica	South Pacific (AgSPc)
A. germinans	RSD-11-10	EF540990	EF540967	EU352185	10.066	-84.966	Punta Morales		Costa Rica	South Pacific (AgSPc)
A. germinans	RSD-ALI-1	DQ469835	EF540974	EU352181	24.133	-110.433	La Paz	Baja California	Mexico	North Pacific (AgNPc)
A. germinans	RSD-Ct2	DQ469836	EF540975	EU352180	16.683	-99.95	Chautengo	Guerrero	Mexico	North Pacific (AgNPc)
A. germinans	RSD-PO1T	EU352145	EU352156	EU352169	15.933	-93.8	Tonalá	Chiapas	Mexico	North Pacific (AgNPc)
A. germinans	RSD-PO9T	EU352146	EU352157	EU352170	15.933	-93.8	Tonalá	Chiapas	Mexico	North Pacific (AgNPc)
A. germinans	RSD-Agt1	EU352147	EU352154	EU443253	15.933	-93.8	Tonalá	Chiapas	Mexico	North Pacific (AgNPc)
A. germinans	RSD-Agt10	EU352148	EU352155	EU443252	15.933	-93.8	Tonalá	Chiapas	Mexico	North Pacific (AgNPc)
A. germinans	RSD-C21T	EF540983	EF540947	EU352178	24.916	-112.217	La Cigueña	Chiapas	Mexico	North Pacific (AgNPc)
A. germinans	RSD-APC1-4	DQ469834	EF540976	EU352179	24.916	-112.217	Bahia Magdalena		Mexico	North Pacific (AgNPc)
A. germinans	Mex1	EF136920			23.216	-106.416	Mazatlan		Mexico	North Pacific (AgNPc)
A. germinans	Mex2	EF136921			16.683	-99.95	Chautengo		Mexico	North Pacific (AgNPc)
A. germinans	RSD-Be1	DQ469853	EF540970	EU352195	32.333	-64.75			Bermuda	North Atlantic (AgNAt)
A. germinans	RSD-Db3	DQ469854	EF540971	EU352194	19.316	-69.5			Dominican Republic	North Atlantic (AgNAt)
A. germinans	AgCe6	DQ469850			20.86	-90.4	Celestum		Mexico	North Atlantic (AgNAt)
A. germinans	AgDOM	EF136923			19.96	-69.56			Dominican Republic	North Atlantic (AgNAt)
A. germinans	AgUSA	EF136922			26.266	-82.3		Florida	USA	North Atlantic (AgNAt)
A. germinans	T11	DQ469850			20.2166	-87.4666	Tulum		Mexico	North Atlantic (AgNAt)

A. germinans	RSD-An2-7	DQ469860	EF540969	EU352193	12.36	-5.116	Soyo		Angola	West Africa (AgAfr)
A. germinans	AgB7	DQ469859			12.28	-16.15			Guinea-Bissau	West Africa (AgAfr)
A. germinans	AgGMB	EF136927			13.05	-15.516			Gambia	West Africa (AgAfr)
A. germinans	AgSEN	EF136928			12.583	-16.26	Zinguichor		Senegal	West Africa (AgAfr)
A. germinans	Pg17	DQ469863			9.609	-80.665	Galeta		Panama	South Caribe (AgSCr)
A. germinans	RSD-20-2	DQ469852	EF540968	EU352192	9.55	-79.65	Rio Claro		Panama	South Caribe (AgSCr)
A. germinans	AgGUA1	EF136924			16.35	-62.03			Guadeloupe	South Caribe (AgSCr)
A. germinans	AgGUA2	EF136925			16.33	-61.733			Guadeloupe	South Caribe (AgSCr)
A. germinans	AgMt1	DQ469855			14.066	-61			Martinique	South Caribe (AgSCr)
A. germinans	AgMt2	DQ469856			14.066	-61			Martinique	South Caribe (AgSCr)
A. germinans	AgA5	DQ469857			5.383	-52.833			French Guiana	South Caribe (AgSCr)
A. germinans	N4	DQ469858			5.766	-35.25	Natal	Rio Grande do Norte	Brazil	Natal (AgNTL)
A. germinans	NAT	EF136926			5.7	-35.3	Natal	Rio Grande do Norte	Brazil	Natal (AgNTL)
A. germinans	AgALC (21)	AB860420-B860440	AB860751-AB860771	AB861082-AB861102	-2.40971	-44.4057	Alcântara	Maranhão	Brazil	Alcântara (AgALC)
A. germinans	AgMRJ (24)	AB860441-AB860464	AB860772-AB860795	AB861103-AB861126	-0.70565	-48.4863	Soure	Pará	Brazil	Marajó (AgMRJ)
A. germinans	AgPAa (25)	AB860465AB860489	AB860796-AB860820	AB861127-AB861151	-0.89277	-46.687	Bragança	Pará	Brazil	Pará* (AgPAa)
A. germinans	AgPAb (16)	AB860490-AB860505	AB860821-AB860836	AB861152-AB861167	-1.93916	-46.7214	Bragança	Pará	Brazil	Pará (AgPAb)
A. germinans	AgPNB (24)	AB860506-AB860529	AB860837-AB860860	AB861168-AB861191	-1.93916	-46.7214	Bragança	Pará	Brazil	Pará (AgPAb)
A. germinans	AgPRC (4)	AB860530-AB860533	AB860861-AB860864	AB861192-AB861195	-2.78051	-41.8236	Parnaíba	Piauí	Brazil	Parnaíba (AgPNB)
A. germinans	AgTMD (24)	AB860534-AB860557	AB860865-AB860888	AB861196-AB861219	-3.41269	-39.0571	Paracuru	Ceará	Brazil	Paracuru (AgPRC)
A. germinans	AgALC (21)	AB860420-B860440	AB860751-AB860771	AB861082-AB861102	-8.58974	-35.0645	Tamandaré	Pernambuco	Brazil	Tamandaré (AgTMD)
A. schaueriana Stapf and Leechm. Ex Moldenke										
A. schaueriana	RSD-Sh1	DQ469862	EF540952	EU352166			Macao		Brazil	Natal (AsNTL)
A. schaueriana	RSD-ShG1	EF540986	EF540945	EU352165					Guadeloupe	South Caribe (AsSCr)
A. schaueriana	AsPAR (26)	AB860558-AB860583	AB860889-AB860914	AB861220-AB861245	-2.40971	-44.4057	Alcântara	Maranhão	Brazil	Pará (AsPAR)
A. schaueriana	AsALC (22)	AB860584-AB860605	AB860915-AB860936	AB861246-AB861267	-24.8971	-47.8472	Cananéia	São Paulo	Brazil	Alcântara (AsALC)

A. schaueriana	AsCNN (23)	AB860606-AB860628	AB860937-AB860959	AB861268-AB861290	-27.5678	-48.5189	Florianópolis	Santa Catarina	Brazil	Cananéia (AsCNN)
A. schaueriana	AsFLN (22)	AB860629-AB860650	AB860960-AB860981	AB861291-AB861312	-22.6989	-43.0015	Guapimirim	Rio de Janeiro	Brazil	Florianópolis (AsFLN)
A. schaueriana	AsGPM (24)	AB860651-AB860674	AB860982-AB861005	AB861313-AB861336	-0.82	-46.615	Bragança	Pará	Brazil	Guapimirim (AsGPM)
A. schaueriana	AsPPR (23)	AB860675-AB860697	AB861006-AB861028	AB861337-AB861359	-25.623	-48.355	Ponta do Paraná	Paraná	Brazil	Pontal do Paraná (AsPPR)
A. schaueriana	AsPRC (16)	AB860698-AB860713	AB861029-AB861044	AB861360-AB861375	-3.41269	-39.0571	Paracuru	Ceará	Brazil	Paracuru (AsPRC)
A. schaueriana	AsUBA (21)	AB860714-AB860734	AB861045-AB861065	AB861376-AB861396	-23.49	-45.163	Ubatuba	São Paulo	Brazil	Ubatuba (AsUBA)
A. schaueriana	AsVER (16)	AB860735-AB860750	AB861066-AB861066	AB861397-AB861397	-12.934	-38.6742	Vera Cruz	Bahia	Brazil	Vera Cruz (AsVER)

Taxa, collection code with sample sizes within parenthesis for samples we collected, location (degrees decimal, and geopolitical units) and geographic region (with abbreviation between parentheses) of the samples evaluated using ITS (nDNA), *trn*D-*trn*T and *trn*H (cpDNA) markers.
