# Supplementary material for: Species distribution and introgressive hybridization of two Avicennia species from the Western Hemisphere unveiled by phylogeographic patterns
Source: BMC Evol Biol. 2015 Apr 10;15:61. doi: 10.1186/s12862-015-0343-z (PMC4394560; doi:10.1186/s12862-015-0343-z)
Supplement: Additional file 2: — Intraspecific pairwise genetic structure for A. germinans and A. schaueriana. Pairwise ΦST between samples of A) A. germinans and B) A. schaueriana. The values below the diagonal were obtained using the ITS marker, and the values above the diagonal were obtained using the DTHK marker. The bold and underlined numbers indicate the nonsignificant pairwise ΦST values after 10,000 bootstraps. [file 12862_2015_343_MOESM2_ESM.pdf]

Additional file 2. Intraspecific pairwise genetic structure for *A. germinans* and *A. schaueriana.*

A)	AgNorthPac	AgSouthPac	AgMRJ	AgPAa	AgPAb	AgALC	AgPNB	AgTMD
AgNorthPac	0	0.62011	0.71146	0.62097	0.53751	0.58846	0.88317	0.58822
AgSouthPac	0.12008	0	0.74324	0.67136	0.6134	0.64837	0.86992	0.64548
AgMRJ	0.24291	0.17712	0	0.68928	0.05731	0.06755	0.10638	0.56896
AgPAa	0.43288	0.34072	0.20583	0	0.56636	0.56554	0.80509	0.61726
AgPAb	0.25753	0.18733	0.06627	0.04003	0	0.02684	0.18289	0.48602
AgALC	0.36376	0.28111	0.32617	0.48135	0.31844	0	0.17296	0.52436
AgPNB	0.50875	0.40074	0.46251	0.60427	0.4623	0.02111	0	0.74692
AgTMD	0.50875	0.40074	0.23006	0.62517	0.45982	0.54002	0.67609	0

B)	AsPAR	AsALC	AsPRC	AsVER	AsGPM	AsUBA	AsCNN	AsPPR	AsFLN
AsPAR	0	0.58305	0.54811	0.45358	0.55423	0.57764	0.58832	0.54826	0.46527
AsALC	0.34819	0	0	0.08477	0.01863	0	0	0.0208	0.06977
AsPRC	0.0952	0.4112	0	0.06452	0.0092	0	0	0.01093	0.05402
AsVER	0.51788	0.59724	0.36622	0	0.031	0.0816	0.08788	0.02874	0.00182
AsGPM	0.31557	0.37978	0.19219	0.1411	0	0.01723	0.01997	0.00051	0.01792
AsUBA	0.52768	0.63921	0.38043	0.02725	0.21234	0	0	0.01933	0.06733
AsCNN	0.633	0.71365	0.51747	0.03993	0.27771	0.05683	0	0.02222	0.07216
AsPPR	0.46438	0.53446	0.30882	0.00156	0.08606	0.05325	0.08631	0	0.02694
AsFLN	0.68716	0.77326	0.59863	0.11533	0.34297	0.12541	0.0426	0.15192	0

Pairwise Φ_{ST} between samples of A) *A. germinans* and B) *A. schaueriana.* The values below the diagonal were obtained using the ITS marker, and the values above the diagonal were obtained using the DTHK marker. The bold and underlined numbers indicate the non-significant pairwise Φ_{ST} values after 10,000 bootstraps.
